# Histopathological Spectrum of Endometrium in Abnormal Uterine Bleeding in a Tertiary Care Centre: A Three-Year Retrospective Study

**DOI:** 10.7759/cureus.77542

**Published:** 2025-01-16

**Authors:** Mary Nandini Singh, Athira Sasidharan, Benzy Paul, Sathi Puthen Parambath

**Affiliations:** 1 Pathology, Kunhitharuvai Memorial Charitable Trust Medical College, Kozhikode, IND

**Keywords:** abnormal uterine bleeding, carcinoma, endometrial biopsy, hyperplasia, post-menopausal bleeding

## Abstract

Background: Abnormal uterine bleeding (AUB) is a universally prevalent affliction seen worldwide. However, the diagnosis and treatment of AUB are rarely straightforward due to the multifactorial aetiology, often requiring varied investigations to differentiate between functional and organic pathological lesions. A crucial diagnostic method that is beneficial is a simple endometrial biopsy procedure with an accurate histopathology diagnosis. This interventional procedure in patients suffering from AUB is warranted when medical management fails or if a radiologically suspicious endometrial thickening is seen, especially in perimenopausal and postmenopausal women.

Aim: The present study was undertaken to study the histopathological spectrum of endometrial lesions, to identify the various functional/organic causes of AUB, and to highlight the importance of a definitive histopathology diagnosis in challenging cases. The study also gathered data from AUB patients diagnosed with endometrial hyperplasia/carcinoma on biopsy who underwent a hysterectomy at our institution, with the aim of examining the concordance rate between the initial biopsy diagnosis and the final diagnosis.

Materials and Methods: A retrospective three-year study was conducted in a tertiary care centre with 956 patients presenting with AUB who underwent endometrial sampling, dilation and curettage (D&C)/Pipelle aspiration as a diagnostic work-up.

Results: Of the 956 endometrial samples received, 106 were found to be inadequate for diagnosis, reducing the sample size to 850. Data analysis showed that perimenopausal women had the highest incidence of AUB (379/850; 44.5%). The incidence of functional and organic causes of AUB was 73.9% (628/850) and 26.1% (222/850), respectively. The most common functional cause of AUB was disordered proliferative endometrium (DPE) (235/628; 27.7%), followed by proliferative endometrium with 155/850 cases (18.2%). The most common organic lesion was benign endometrial polyp (102/850; 12%). It was followed by non-atypical hyperplasia (55/850; 6.5%) and then atypical endometrial hyperplasia/endometrial intraepithelial neoplasia (AEH/EIN) (41 cases, or 4.8%). Of the 22 cases of endometrial carcinomas, 81.8% were of the endometrioid type, followed by 9.2% cases of carcinosarcoma, and the remaining were clear cell carcinoma (4.5%) and serous carcinoma (4.5%).

Conclusion: Endometrial sampling, D&C/Pipelle aspiration is an essential gynaecological investigation in the evaluation of AUB. An accurate histopathology diagnosis, especially in endometrial malignancies, brings clarity and sets the stage for effective treatment strategies.

## Introduction

Abnormal uterine bleeding (AUB) is defined as any bleeding from the genital tract that is a deviation from the normal in frequency, cyclicity, duration, or quantity. AUB accounts for approximately 25-33% of outpatient visits to gynaecologists [1]. It is one of the most common disorders seen in women of all age groups, starting from adolescence (due to anovulatory cycles) to worrisome bleeding in postmenopausal age (possibly due to precursor lesions like atypical hyperplasia or even carcinoma) [1]. The onset of AUB typically occurs in the fourth to fifth decade (perimenopausal age group), coinciding with the body's transition to menopause [1]. The clinical presentation of AUB can vary among patients with heavy menstrual bleeding (HMB), heavy and prolonged menstrual bleeding (HPMB), irregular bleeding, intermenstrual bleeding, postcoital bleeding, and postmenopausal bleeding (PMB) [1].

The International Federation of Gynecology and Obstetrics (FIGO) has introduced a classification system in 2011 to categorise AUB (PALM-COEIN). The terms polyp, adenomyosis, leiomyoma, malignancy (PALM), and hyperplasia describe problems with the structure of the uterus, while coagulopathy, ovulatory disorders, endometrial, iatrogenic, and not otherwise classified (COEIN) describe problems with the function of the uterus that lead to AUB [1-3]. In the perimenopausal setting, hyper-estrogenism and anovulatory cycles are a common cause of AUB [4,5].

AUB can be a chronic cause of morbidity in many patients, leading to significant disruptions in their quality of life due to complex social and interpersonal issues. If left unresolved, it can lead to the development of severe anemia [6]. Endometrial biopsy is done when medical management fails or if the patient has a thickened endometrium and/or risk factors for endometrial cancer [6]. There are various methods for sampling the endometrium, including dilation and curettage (D&C), endometrial biopsy, hysteroscopy-guided endometrial biopsy, and Pipelle aspiration. Pipelle aspiration is considered a safe and simple alternative to D&C, being an outpatient procedure with reasonably acceptable diagnostic accuracy [7].

The aim of this study was to analyse the histomorphological spectrum of endometrial lesions in AUB and subclassify them according to functional or organic causes as per World Health Organization (WHO) Classification of Tumours of Female Genital Tumours, fifth edition, and Blaustein’s Pathology of the Female Genital Tract, seventh edition [8,9]. We studied the incidence/frequency of endometrial lesions (functional or organic) in different age group settings (reproductive, perimenopausal, and postmenopausal) in women with AUB. We also analysed the data obtained from AUB patients diagnosed to have endometrial hyperplasia/carcinoma on biopsy and who underwent subsequent hysterectomy at our institution. This was done to analyse the concordance rate of the initial endometrial biopsy and total hysterectomy.

## Materials and methods

Source of data

This was a three-year retrospective record based study conducted on women presenting with AUB (from July 2020 to June 2023). The Department of Pathology at Kunhitharuvai Memorial Charitable Trust Medical College, Kozhikode, conducted the study with approval from the Institutional Ethics Committee.

Inclusion criteria

Endometrial samples of women with a clinical diagnosis of AUB who underwent an endometrial biopsy procedure (D&C/Pipelle aspiration) at the institution.

Exclusion criteria

The study excluded endometrial samples of women with bleeding from cervical and vaginal diseases, haemostatic disorders, pregnancy complications such as missed abortions, or suspected molar pregnancies.

Methods of data collection

A record-based retrospective analysis of 956 endometrial samples (D&C/Pipelle aspiration) collected from AUB patients was done. We obtained the patient's clinical history and relevant investigations from the patient's requisition forms. We retrieved the hematoxylin and eosin- (H&E-) stained histopathology slides, re-examined them, and confirmed the final histopathological diagnosis. Three age categories were chosen: reproductive (18-40 years), perimenopausal (41-50 years), and postmenopausal (≥51 years) and correlated with histopathological pattern seen. We re-examined the slides of AUB patients who underwent subsequent hysterectomy at our institution to confirm the final histopathological diagnosis. Categorical variables were presented in number and percentage (%), and continuous variables were presented as mean ± SD and median. We entered the data into an Microsoft Excel spreadsheet to generate tables. Pearson’s Chi-square test (p value) was used to determine the significance of the association between age and functional versus organic causes of AUB.

## Results

**Figure 1:**
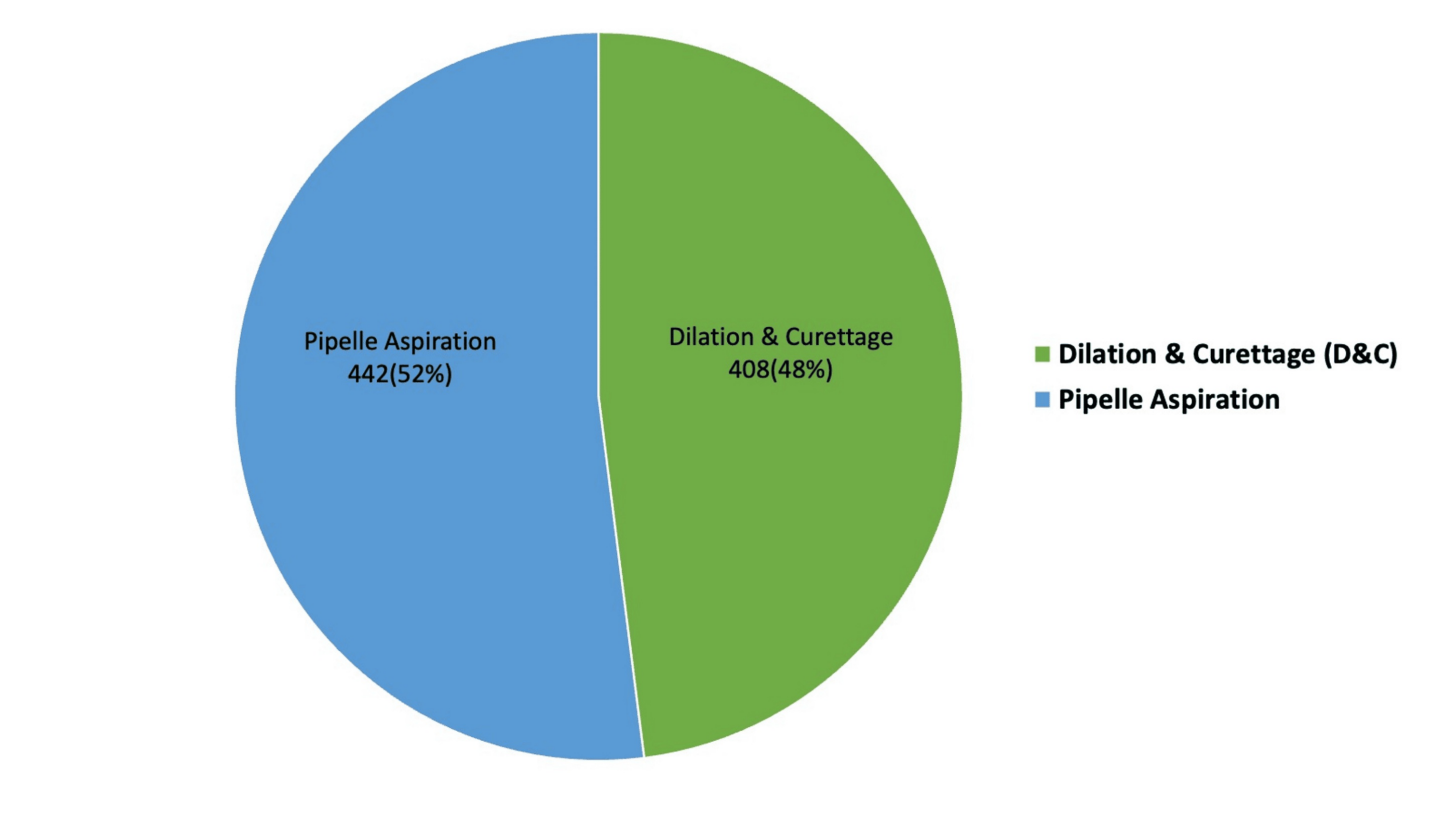
Method of endometrial sampling employed (n=850)

**Figure 2:**
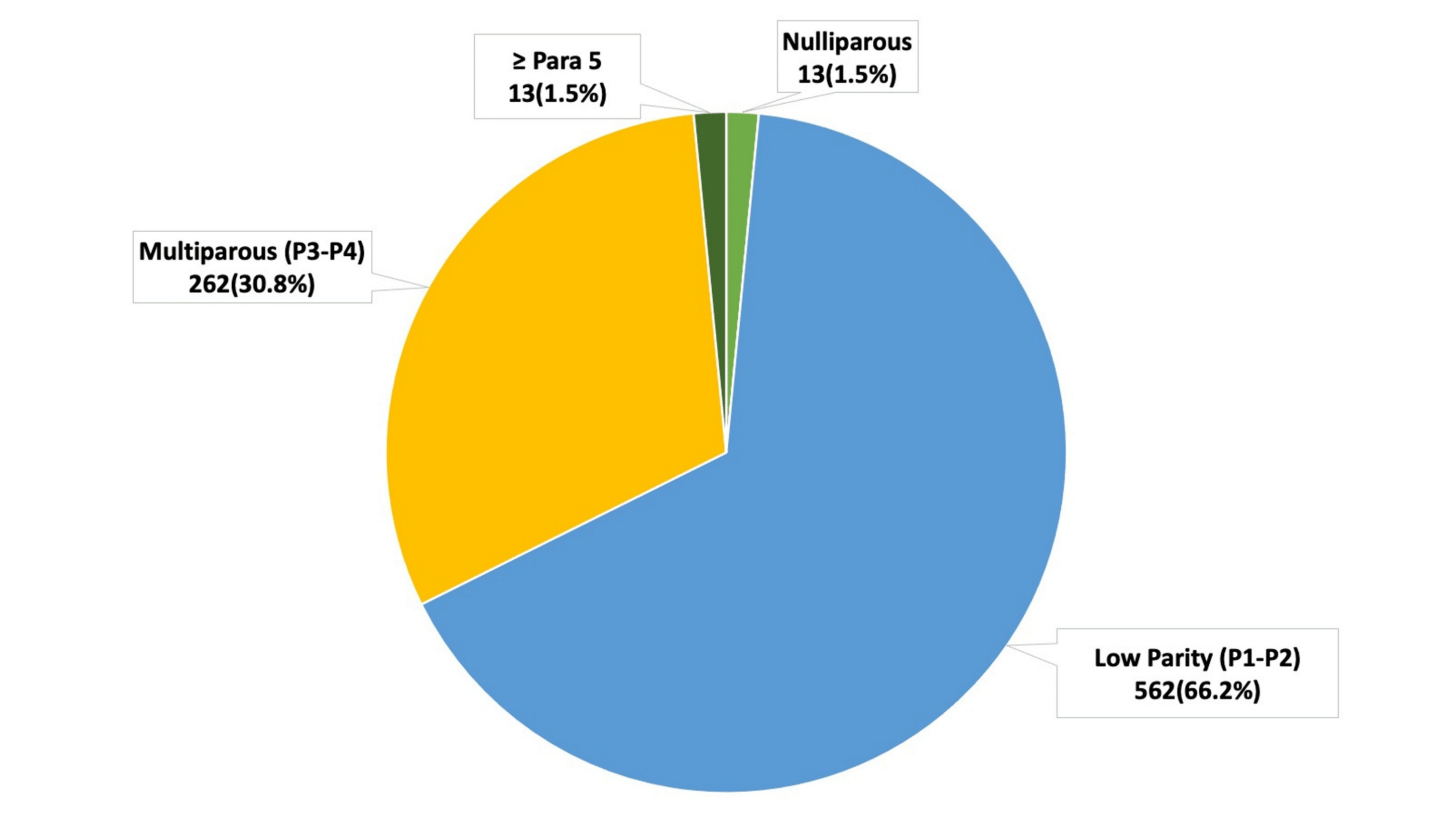
Distribution of cases according to parity (n=850)

In this study, we analysed a total of 956 AUB cases to establish an endometrial biopsy diagnosis. Figure 1 illustrates the endometrial sampling method employed. We deemed 106 of the 956 samples inadequate for diagnosis, resulting in a reduction in the sample size to 850. The age of the patients ranged from 23 to 79 years. The mean age calculated was 46.9 years, and the median age was 46 years. The youngest patient (23 years) presented with a benign endometrial polyp, while the oldest patient (78 years) had features suspicious for endometrial carcinoma. We categorised the data into three age groups: the reproductive age group of 18-40 years (n = 271), the perimenopausal age group of 41-50 years (n = 379), and the postmenopausal age group of ≥51 years (n = 200), as seen in Table 1. The perimenopausal age group of 41-50 years had the highest incidence of AUB (379/850; 44.5%). Analysis of parity as seen in Figure 2 showed the highest incidence of AUB among the low parity group (562/850; 66.2%), followed by the multiparous category with 30.8% of cases (262/850), the grand multipara with 1.5% of cases (13/850), and the nulliparous group with 1.5% of cases (13/850).

**Table 1:** Distribution of AUB cases according to age groups AUB: Abnormal uterine bleeding

Age of patient (Years)	Number of AUB cases (n)	Percentage
18-40	271	32.0%
41-50	379	44.5%
≥51	200	23.5%
Total no. of AUB cases	850	100%

**Figure 3:**
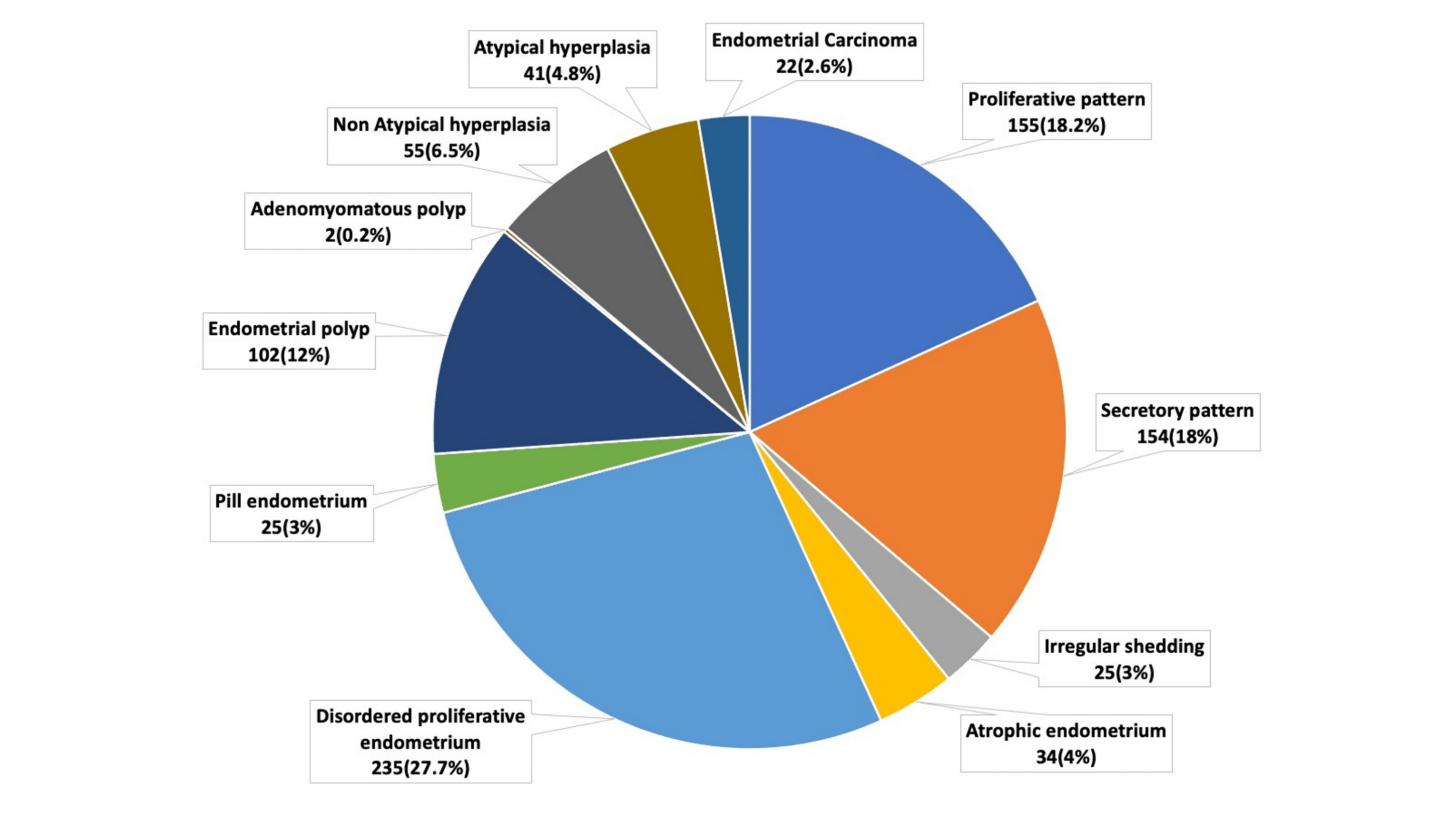
Distribution of cases of AUB according to the histopathological patterns (n=850) AUB: Abnormal uterine bleeding

**Figure 4:**
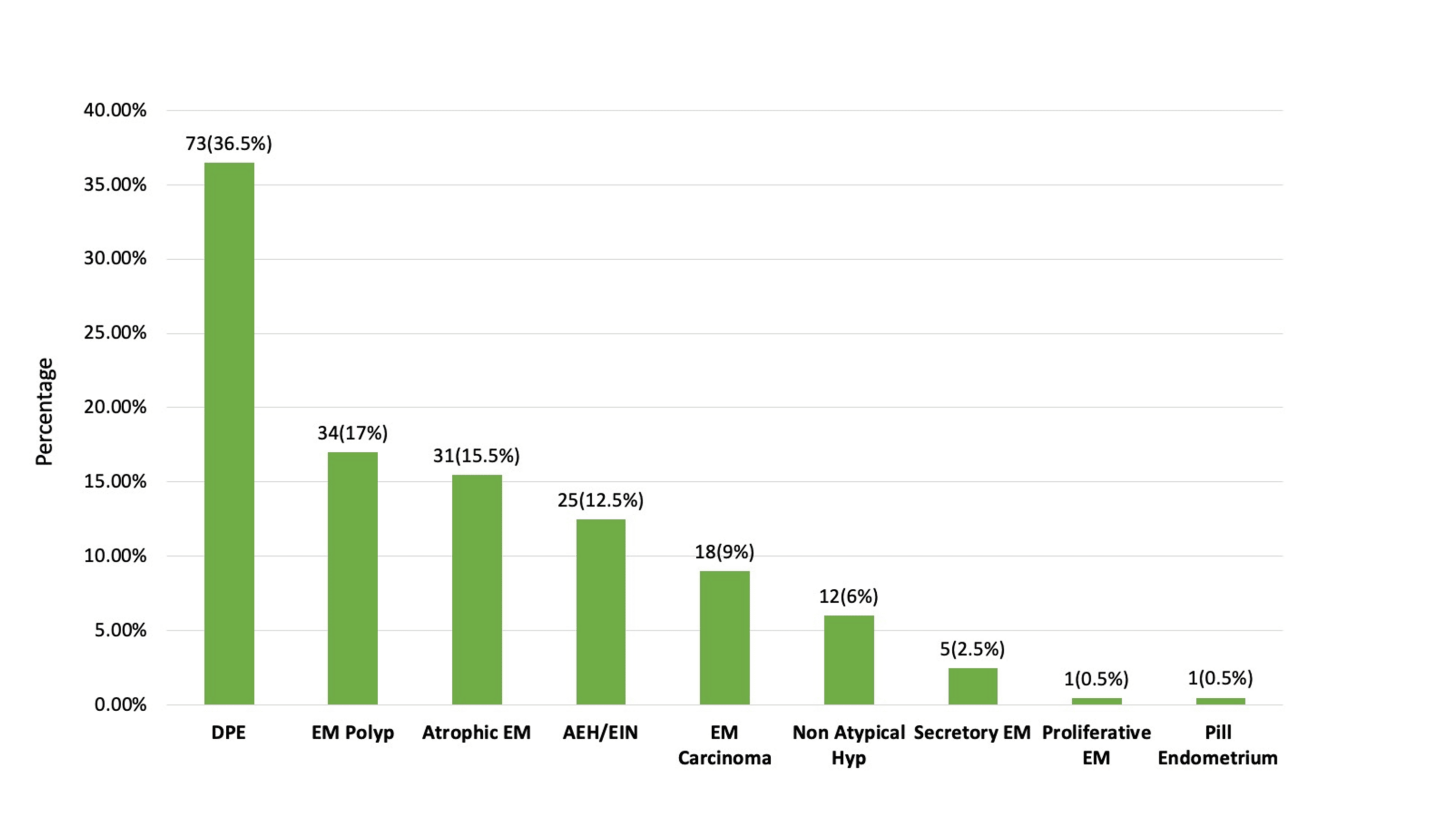
Histopathological spectrum of endometrial lesions seen in PMB (n=200) AEH/EIN: Atypical endometrial hyperplasia/Endometrial intraepithelial neoplasia; DPE: Disordered proliferative endometrium; EM: Endometrium/endometrial; Hyp: Hyperplasia; PMB: Postmenopausal bleeding

**Figure 5:**
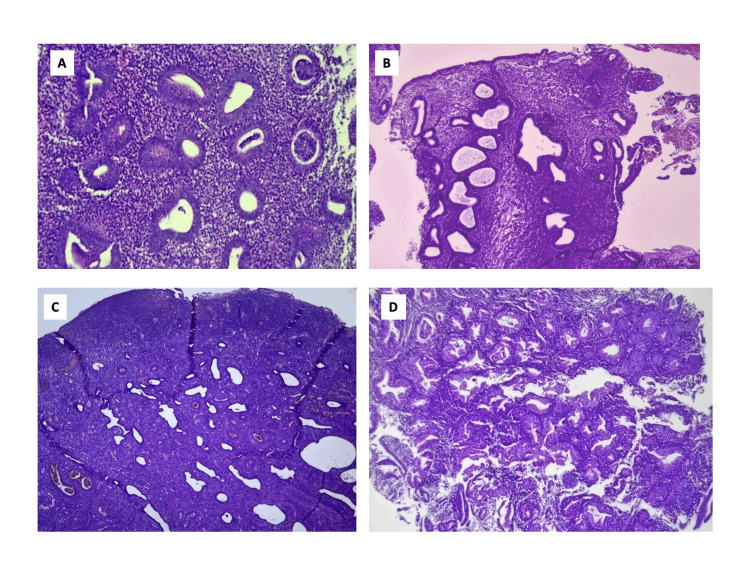
Photomicrograph of various endometrial lesions in AUB 5A: Proliferative endometrium, 40 years (H&E, 10x); 5B: DPE, 49 years (H&E, 4x); 5C: Benign endometrial polyp, 45 years (H&E, 4x); 5D: AEH, 52 years (H&E, 4x) AEH: Atypical endometrial hyperplasia; AUB: Abnormal uterine bleeding; DPE: Disordered proliferative endometrium; H&E: Hematoxylin and eosin

**Figure 6:**
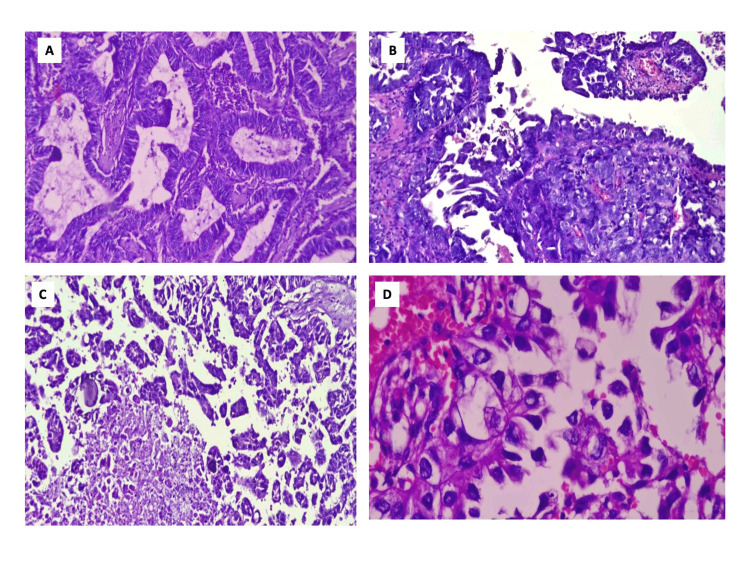
Photomicrograph of various neoplastic endometrial lesions in AUB 6A: Endometrioid adenocarcinoma, 51 years (H&E, 40x); 6B: Carcinosarcoma endometrium, 52 Years (H&E, 40x); 6C: Serous carcinoma endometrium, 64 Years (H&E, 10x); 6D: Clear cell carcinoma endometrium, 65 Years (H&E, 40x) AUB: Abnormal uterine bleeding; H&E: Hematoxylin and eosin

The functional and organic causes of AUB were analysed and compared with the different age groups, as seen in Tables 2, 3. The distribution of 850 cases of AUB is depicted in Figure 3. The disordered proliferative endometrium (DPE) was the most common functional cause of AUB (235; 27.7%). The age category of perimenopausal women was found to have the highest concentration of DPE cases (129; 55%). Analysis of the 200 postmenopausal women with AUB also showed DPE as the most common lesion (73; 36.5%) (Figure 4). In Figure 5B, a photomicrograph of DPE is presented. Proliferative endometrium as shown in Figure 5A is the second most common functional pattern seen (155/850; 18.2%). 34/850 AUB cases (4.0%) showed atrophic changes in the endometrium. We observed an irregular shedding pattern in 25/850 AUB cases (3.0%). 25/850 AUB cases (2.9%) showed extensive decidualisation of the endometrium (pill or exogenous hormonal effect on endometrium). Benign endometrial polyps were the most common organic cause of AUB, with 102/850 cases (12%), as shown in Figure 5C. In addition, we observed two cases of benign adenomyomatous polyps. Analysis of the 96/850 cases of endometrial hyperplasia showed 55 cases of non-atypical hyperplasia and 41 cases of atypical endometrial hyperplasia/endometrial intraepithelial neoplasia (AEH/EIN) as shown in Figure 5D. The 22/850 cases of endometrial carcinomas included 18 cases of the endometrioid type (81.8%) and two cases of carcinosarcoma. The remaining two were one case of serous carcinoma and one case of clear cell carcinoma. The photomicrographs of the neoplastic endometrial lesions are shown in Figures 6A to 6D. Functional causes of AUB accounted for 73.9% of the cases (628/850), while organic causes accounted for 26.1% (222/850). The Chi-square test showed a p-value of 0.001, when the association between age and functional versus organic causes of AUB was studied, which is statistically significant (Table 3).

**Table 2:** Distribution of histopathological patterns according to age groups in patients with AUB AUB: Abnormal uterine bleeding; DPE: Disordered proliferative endometrium

S. No.	Functional versus organic causes	Histopathologic diagnosis	18-40 years	41-50 years	≥ 51 years	Total no. of cases
1	Functional causes	DPE	33 (14%)	129 (55%)	73 (31%)	235
2		Proliferative pattern	78 (50.3%)	76 (49.1%)	1 (0.6%)	155
3		Secretory pattern	99 (64.3%)	50 (32.5%)	5 (3.2%)	154
4		Atrophic endometrium	0	3 (8.8%)	31 (91.2%)	34
5		Irregular shedding	12 (48%)	13 (52%)	0	25
6		Pill endometrium	6 (24%)	18 (72%)	1 (4%)	25
7	Organic causes	Endometrial polyp	24 (23.6%)	44 (43%)	34 (33.4%)	102
8		Non-atypical hyperplasia	14 (25.4%)	29 (52.6%)	12 (22%)	55
9		Atypical hyperplasia	2 (4.8%)	14 (34.2%)	25 (61%)	41
10		Endometrial carcinoma	1 (4.6%)	3 (13.6%)	18 (81.8%)	22
11		Adenomyomatous polyp	2 (100%)	0	0	02
	Total no. of cases		271	379	200	850

**Table 3:** Comparison between age and functional versus organic causes of AUB AUB: Abnormal uterine bleeding Chi-square test (p-value)

Cause of AUB	18-40 years (Reproductive)	41-50 years (Perimenopausal)	≥ 51 years (Postmenopausal)	Total no. of cases	p value
Functional	228	289	111	628	0.001
Organic	43	90	89	222	
Total	271	379	200	850	

There were 200 cases of PMB. The histopathological analysis of postmenopausal women revealed DPE as the most common functional pattern (73/200; 36.5%), followed by 34 cases of endometrial polyps (17.0%), 31 cases of atrophic endometrium (15.5%), 25 cases of AEH/EIN (12.5%), 18 cases of endometrial carcinoma (9.0%), and 12 cases of non-atypical hyperplasia (6.0%) (Figure 4). We collected data of women who underwent hysterectomy in our institution and checked the concordance rate with the endometrial biopsy diagnosis, which is tabulated in Table 4.

**Table 4:** Comparison between endometrial biopsy and hysterectomy in AUB patients AUB: Abnormal uterine bleeding; EM: Endometrial/endometrium

EM biopsy diagnosis	Total no. of EM biopsy cases	Cases that underwent hysterectomy at our institution	Concordance with biopsy	Post-hysterectomy, change in final diagnosis
Non-atypical EM hyperplasia	55	2	2	0
Atypical EM hyperplasia	41	6	4	2
Endometrial carcinoma	18	14	14	0

## Discussion

Women typically present with AUB in the perimenopausal age group [1]. Rendering an accurate histopathology diagnosis in AUB patients can sometimes be quite challenging, as the endometrium is a hormonally responsive dynamic tissue with a rapidly changing landscape causing much inter-observer variability [1]. In perimenopausal and postmenopausal settings, where anovulatory cycles come into play and neoplasms can arise, the pathologist plays a significant role in detecting precursor lesions of the endometrium and in confirming or ruling out a neoplastic process [4].

In our study of 956 AUB cases, we assessed 106 endometrial samples to be inadequate for diagnosis. The Pipelle aspiration method showed a higher incidence of inadequate to scanty tissue (sampling inadequacy) (67/956; 7.0%) compared to the traditional D&C method (39/956; 4.0%). In the current scenario, the most popular and simple method for endometrial sampling is undoubtedly Pipelle aspiration. However, the disadvantages of Pipelle aspiration include the limited yield, particularly in postmenopausal women, and there is an inadvertent possibility of missing focal lesions. Because of this, the traditional D&C method must be used after Pipelle aspiration if there is a clinical suspicion of malignancy and the Pipelle report is not diagnostic, or if there are some concerning features on the Pipelle sample that are not enough to make a definite diagnosis of malignancy.

The perimenopausal age group (44.5%) showed a peak incidence of AUB in our population. This is comparable to the findings of earlier studies by Kafle et al. (42.77%) and Doraiswami et al. (33.5%) [5,10]. The percentages seen in Mune et al. (42%) and Desai et al. (42.8%) are also similar to our study [11,12]. Studies that showed variation in incidence in the perimenopausal age group when compared to our study was Vijayaraghavan et al., which had a higher incidence of 56.3% [13]. Perimenopausal AUB is commonly seen and can arise from a complex interplay of hormonal fluctuations and structural abnormalities like uterine leiomyomas or endometrial polyps. Perimenopausal women are also at increased risk for oestrogen-driven endometrial hyperplasia and have a heightened risk for endometrial cancer [14]. Our study found that women with low parity were more likely to have AUB (66.2%) than women who were multiparous (30.8%), grand multiparous (1.5%), or those women who have never been pregnant (1.5%). This is in contrast with other studies in which AUB was more prevalent in multiparous groups [15,16]. Global cultural trends, along with the high literacy rate and economic status of Kerala, with a higher proportion of small nuclear families, could partially explain the contrasting data in relation to parity.

Histopathological examination of the endometrium showed both functional endometrial lesions and definite organic pathology. As age advances, an anticipated upward trend of organic lesions of endometrium is seen with Chi Square test showing a statistically significant p value of 0.001. The comparison of functional versus organic causes of AUB showed that DPE was the most common functional cause of AUB (235/628, 27.7%). The incidence of DPE in other studies was found to be lower (20.53% and 19.38%) by Doraiswami et al. and Desai et al. [10,12]. In our study, the perimenopausal age group had the highest rate of DPE (55%), followed by the postmenopausal age group (31%). This data is comparable to the study done by Doraiswami et al. (41-50 years; 47.6%) [10]. However, the incidence of DPE in a study by Damle et al. was much lower at 15.90% [17].

DPE is a pattern often associated with AUB in the context of anovulatory cycles. It consists of irregularly, variably shaped glands, some cystically dilated, embedded in a proliferative stroma. Although DPE is considered a normal finding in the perimenopausal age group, in postmenopausal patients with AUB, it may represent an indicator of impending hyperplasia, and such patients should be followed up. Proliferative endometrium was the second most common functional cause of bleeding, accounting for 155/850 cases (18.2%). Abnormal bleeding, particularly in the proliferative phase, is most likely oestrogen driven due to anovulatory cycles, while secretory phase dysfunction primarily stems from ovulatory DUB with insufficient progesterone levels, which leads to endometrial instability and subsequent endometrial breakdown. The atrophic and weakly proliferative endometrium is normal in both prepubertal and postmenopausal settings [18]. Atrophic endometrium was seen in 31 cases with PMB (15.5%). Our study found a reduced incidence of endometrial atrophy compared to Gredmark et al., which identified it in 50% of cases [19]. 

Among the organic causes of AUB, benign endometrial polyp is the most common pathology seen with 102/850 cases (12%). This incidence rate is similar to the findings of Doraiswami et al. (11.2%) and Parmar et al. (10.78%) [10,20]. Our study found that the perimenopausal age group had the highest rate of benign endometrial polyps (43%). This may be because perimenopausal women have higher levels of estrogen, which causes the endometrial basalis to grow excessively and form endometrial polyps [21].

The next most common cause of organic pathology leading to AUB was non-atypical hyperplasia with 55/850 cases (6.5%), followed by AEH/EIN with 44/850 cases (4.8%). This was almost similar to other studies done by Doraiswami et al. [10]. In a large-scale study (n=4247) done in Turkey, the incidence of endometrial hyperplasia was seen as 9.68%, which is almost comparable to our study [22]. In our study, the highest incidence of non-atypical hyperplasia (29/55; 52.6%) was seen in the 41-50 age group, and the highest incidence of atypical hyperplasia was seen in the ≥ 51 years age group (25/41; 61%). Literature shows that AEH/EIN is more commonly diagnosed in the postmenopausal age group, correlating with our study [23]. Chronic anovulation, obesity, which further elevates estrogen levels, and exogenous estrogen therapy can all lead to AEH/EIN. The risk of concurrent carcinoma in patients with AEH/EIN can be as high as 42.6%, and therefore AEH/EIN should be managed with hysterectomy for women who have completed childbearing [23].

In our study, the yield of a malignant pathology (endometrial carcinoma) was low (22/850; 2.6%). Some studies, such as Manjari et al., conducted at an oncology referral center, reported a higher incidence at 15%, which can be explained as due to referral bias [24]. In our study, the postmenopausal age group showed the highest incidence of endometrial carcinoma (18/200; 9.0%), which underscores the importance of a detailed evaluation of all patients with PMB so as to detect malignancy as early as possible, leading to successful intervention and management [19,23].

Follow-up of the precancerous/neoplastic AUB patients for the final hysterectomy diagnosis was disappointing. Only 22 of the 66 cases with atypical hyperplasia/suspicious for carcinoma underwent hysterectomy in our institution. Of the six cases of the AEH/EIN category who underwent hysterectomy, only two cases exhibited minimal microscopic myometrial invasion, indicating invasive carcinoma (less than half of myometrium; FIGO stage 1A). Only 14 out of the 22 cases with an endometrial biopsy diagnosis of endometrial carcinoma underwent a hysterectomy at our institute. All 14 patients who underwent hysterectomy showed a morphology consistent with carcinoma with myometrial invasion, thereby confirming the initial biopsy diagnosis. Two patients with non-atypical hyperplasia also underwent hysterectomy. No evidence of endometrial atypia or malignancy was detected in them. One of the non-atypical hyperplasia patients was a 61-year-old female with a functional adult Granulosa cell tumour of the ovary causing hyperestrogenism and subsequent uterine bleeding [25]. A comparison chart with other similar studies is shown in Table 5.

**Table 5:** Comparative study of the results of present study with others AUB: Abnormal uterine bleeding; DPE: Disordered proliferative endometrium; EM: Endometrium; Hyp: Hyperplasia

	Present study	Doraiswami et al. [10]	Mune et al. [11]	Desai et al. [12]	Vijayaraghavan et al. [13]
Total no. of cases	850	409	212	98	160
Type of bleeding	AUB	AUB	AUB	AUB	AUB
Most common age group	41-50 (44.5%)	41-50 (33.5%)	41-50 (42.0%)	41-50 (42.85%)	41-50 (56.3%)
Functional causes	628 (73.9%)	-	137 (64.6%)	62 (63.25%)	104 (65.0%)
Organic lesions	222 (26.1%)	-	75 (35.4%)	36 (36.75%)	56 (35.0%)
Common histopathologic diagnosis	DPE (27.7%)	DPE (20.53%)	DPE (13.7%)	DPE (19.38%)	DPE (7.5%)
	Proliferative pattern (18.2%)	-	Proliferative pattern (27.8%)	Proliferative pattern (20.4%)	Proliferative pattern (35%)
	EM Polyp (12%)	EM Polyp (11.24%)	EM Polyp (8.0%)	EM Polyp (8.16%)	EM Polyp (4.4%)
	Atrophic EM (4.0%)	Atrophic EM (2.4%)	Atrophic EM (2.4%)	Atrophic EM (3.1%)	Atrophic EM (0.97%)
	Chronic endometritis (0.0%)	Chronic endometritis (4.1%)	Chronic endometritis (2.4%)	Chronic endometritis (1.03%)	Chronic endometritis (5.36%)
Frequency of hyperplasia	Non-atypical hyp (6.5%)	Non-atypical hyp (4.16%)	Non-atypical hyp (20.3%)	Non-atypical hyp (15.3%)	Non-atypical hyp (20.6%)
	Atypical hyp (4.8%)	Atypical hyp (2.0%)	Atypical hyp (2.0%)	Atypical hyp (6.12%)	Atypical hyp (5.6%)
Frequency of malignancy	Carcinoma (2.6%)	Carcinoma (4.4%)	Carcinoma (2.3%)	Carcinoma (5.1%)	Carcinoma (1.25%)

The limitations of this study are its retrospective design, limited clinical data regarding the type and duration of bleeding, significant family history, limited radiological data (i.e., ultrasound sonography (USG) findings of endometrial thickness and uterine size correlation), and assessment of risk factors. The other limiting factor seen was lack of follow-up due to the moderately high attrition rates, post-endometrial biopsy (in cases of atypical hyperplasia/carcinoma) for further treatment in specialised oncology referral centres.

## Conclusions

AUB can occur at any age in various forms and has different modes of presentation. A multipronged approach comprising clinical examination, ultrasonography, and endometrial histopathology diagnosis is essential in elucidating a challenging case to optimise treatment and plan further management. In our study, the peak incidence of AUB cases was seen in the perimenopausal age group. We found that organic endometrial pathology, such as atypical hyperplasia and carcinomas, is more common in the perimenopausal and postmenopausal age groups, underscoring the importance of timely endometrial biopsy for the early detection of organic lesions.
